# Engineering of Edge-Enriched Nitrogen-Doped Porous Carbon as a High-Performance Metal-Free Catalyst for Acetylene Hydrochlorination

**DOI:** 10.3390/nano16090568

**Published:** 2026-05-06

**Authors:** Zhenzhen Zhang, Dashuai Zhang, Yalei Hao, Guangzong Fang, Xingyun Li, Jian Qi

**Affiliations:** 1 Institute of Materials for Energy and Environment, College of Materials Science and Engineering, Qingdao University, Qingdao 266071, China; henzhenzhang123@163.com (Z.Z.); haoyalei_2026@163.com (Y.H.); 2 Shandong Provincial Key Laboratory of Monocrystalline Silicon Semiconductor Materials and Technology, College of Chemistry and Chemical Engineering, Dezhou University, Dezhou 253023, China; zhangdashuai@dzu.edu.cn; 3 State Key Laboratory of Catalysis, Dalian Institute of Chemical Physics, Chinese Academy of Sciences, Dalian 116023, China; fgzong@dicp.ac.cn; 4 State Key Laboratory of Biopharmaceutical Preparation and Delivery, Institute of Process Engineering, Chinese Academy of Sciences, Beijing 100190, China

**Keywords:** acetylene hydrochlorination, N doping, co-polymerization, metal-free catalyst

## Abstract

The development of efficient catalysts for acetylene hydrochlorination is critical for replacing the industrially prevalent mercury chloride catalysts. Herein, a defective nitrogen-doped carbon material (NC-APT) is engineered via a facile co-polymerization of pyrrole, aniline, and thiophene, followed by a controlled calcination procedure. This co-polymerization strategy introduces abundant structural defects compared to mono-polymerization processes, primarily due to the lattice mismatch and steric hindrance between the distinct monomers, which disrupts the regularity of the polymer chain and prevents graphitic ordering. The resulting NC-APT catalyst features a high specific surface area of 375.7 m^2^·g^−1^ and a substantial nitrogen dopant content of 14.4%, with 81% of the nitrogen existing as catalytically active edge structures (pyrrolic and pyridinic N). Consequently, the catalyst delivers exceptional performance, achieving 92% acetylene conversion at 220 °C with a C_2_H_2_ gas hourly space velocity (GHSV) of 80 h^−1^. This performance significantly outperforms many reported metal-free counterparts and rivals that of traditional metal-based catalysts. This work offers new insights into the rational design of carbon-based, metal-free catalysts through monomer mismatch engineering.

## 1. Introduction

Polyvinyl chloride (PVC), as one of the most important plastics, finds wide applications in industry and daily life due to its low cost and excellent performance. China is the world’s largest producer and consumer of PVC [1]. About 27 million tons of PVC were produced in China in the year 2024, accounting for more than 50% of global PVC production capacity [2]. Vinyl chloride monomer (VCM), the monomer used to produce PVC, is mainly industrially produced by two routes, including coal-based acetylene hydrochlorination and oil-based ethylene oxychlorination [3]. Particularly, the acetylene involved hydrochlorination process (C_2_H_2_ + HCl → C_2_H_3_Cl) is preferred for counties rich in coal, such as China and India, etc. [4]. However, mercuric chloride (HgCl_2_) loaded on activated carbon is still used as an industrial catalyst for this reaction. The lethal toxicity of HgCl_2_ volatility makes it a serious threat to human health and the ecological environment. The United Nations has reached an agreement in the Minamata Convention for the prohibition of the continued use of mercury [5,6,7]. Therefore, there is an urgent need for the development of green and efficient mercury-free catalysts.

Hutchings et al. first demonstrated the high activity of AuCl_3_ in acetylene hydrochlorination. In addition, Ru-, Pd- and Pt-based catalysts have also shown excellent catalytic performance [8,9,10]. However, the prohibitive cost and limited stability of noble metal catalysts have hindered their widespread practical application. With the encouraging advancement in carbon nanotechnology, great potential was unlocked by applying defective carbon as a metal-free catalyst for acetylene hydrochlorination [11,12]. Notably, N-doped carbon (NC) materials, possessing a unique electronic structure, high-density N-dopant defects and tunable surface properties, has been attracted extensive attention to serve as high-performance catalysts [13,14,15,16]. The mechanistic study by our previous work demonstrated that the pyrrolic N-structure may be the active site for driving the acetylene hydrochlorination reaction, following the Eley–Rideal (E–R) mechanism [17]. This has also been supported by other reports, which showed that the catalytic activity is closely correlated to the pyrrolic N-amount [18,19,20]. However, Chao et al. reported that carbon atoms adjacent to the pyridine N atom in ZIF-8-derived NC are more active for acetylene activation [21]. Qiao et al. elucidated that the pyridinic nitrogen sites with adjacent hydrogen atoms can effectively reduce the reaction energy barrier of acetylene hydrochlorination and boost the catalytic performance [22]. A study by Pérez-Ramiŕez et al. suggested that both pyrrolic and pyridinic N sites are the catalytic actives, which are responsible for activating C_2_H_2_ and HCl, respectively [23]. Despite the above dispute on the real active center, a consensus is reached that engineering a highly porous NC with more exposed N dopant (pyrrolic/pyridinic N) sites could be beneficial for catalytic reactivity improvement.

Recently, N-containing organic polymers (e.g., polypyrrole, polyaniline, polythiophene, etc.) as precursors to produce advanced NC materials have been attracted wide attention [24,25,26]. The superiority of this polymer-derived NCs lies in the high N-doping amount derived from the self-provided N species, and the porous structure induced by the release of volatiles during the heating procedure [27,28,29]. For example, Tao et al. used a simple chemical oxidative polymerization method to produce polyaniline, which was further converted to a microporous NC after simple calcination with abundant pyridinic and pyrrolic N dopants [30]. Meanwhile, polypyrrole obtained from the oxidative polymerization of pyrrole could be conveniently transformed into porous NC with a high N content by the thermal-driven decomposition, which was active for the acetylene hydrochlorination reaction [31,32,33]. Based on the above work and utilizing the common persulfate oxidant-induced polymerization process, we tried to engineer an enhanced edge-N-exposed carbon material by the co-polymerization of pyrrole and aniline. It is anticipated that this co-polymerization could introduce more defects compared with the mono-polymerization process due to the structure mismatch between the distinct monomer units (pyrrole with five-membered ring) and aniline (a benzene ring attached to an amine). Furthermore, an appealing chance could be provided to continuously intervene in the co-polymerization process by leveraging the properties of thiophene, which could offer steric hindrance to disrupt the regularity of the polymer chain and reduce the polymer size [34]. The volatile S species could also bring the oxygen away in the form of SO_x_, thus helping increase the porosity and preserving more N dopants in the final carbon material. Meanwhile, there will be minor S-doping at the edge of the NC, which may facilitate creating a more defective structure [35,36].

Herein, we developed a unique ternary co-polymerization process integrating pyrrole, aniline and thiophene in a one pot synthesis to produce a highly porous NC enriched with edge-N dopants. The defective structure was systematically characterized, and its effective metal-free catalytic properties were investigated for the acetylene hydrochlorination reaction. This study could set an important paradigm of the polymerization-process orchestration for the engineering of a high-performance NC catalyst.

## 2. Experimental

### 2.1. Materials

Aniline (C_6_H_7_N, 99%), thiophene (C_4_H_4_S, 99%), Triton X-100 ((C_2_H_4_O)_n_C_14_H_22_O, 99%), pyrrole (C_4_H_5_N, 99%) and ammonium persulfate ((NH_4_)_2_S_2_O_8_, 99.0%) were purchased from Macklin (Shanghai, China). Acetylene gas (99.99%) and hydrogen chloride (99.99%) were purchased from Yantai Deyi Gas Co., Ltd. (Yantai, China). All materials were used as received without further purification.

### 2.2. Catalyst Preparation

In a typical synthesis, Triton X-100 (0.24 g) was dissolved in 200 mL of deionized (DI) water, which was mechanically stirred for 10 min. Then, aniline (1.5 mL), pyrrole (1.16 mL) and thiophene (0.6 mL) were added to the above solution, separately. After stirring ultrasonically for 2 h, a 1.5 M of ammonium persulfate solution was rapidly added to the solution, which was further stirred for 10 h at 0 °C to trigger the polymerization. Then, the solid product was centrifuged and washed with DI water and ethanol, repeatedly, followed by drying overnight at 60 °C. Finally, the dried powders were calcined at 600 °C for 2 h in a flowing Ar atmosphere to obtain NC-APT. For comparison, NC-AP was prepared following the same conditions as NC-APT but in the absence of thiophene. NC-A and NC-P were synthesized with aniline or pyrrole as the monomer, respectively.

### 2.3. Catalytic Performance Evaluation

The acetylene hydrochlorination reaction was performed in a fixed-bed micro-reactor. Specifically, 0.8 g of the catalyst was added in the middle of a quartz tube (with a radius of 0.3 cm). Before the reaction, the sample was purged with N_2_ for 1 h. Then, a C_2_H_2_/HCl mixture (volume ratio 1:1.2) was fed into the reactor with a C_2_H_2_ gas hourly space velocity (GHSV) of 120 h^−1^ at a reaction temperature of 200 °C and a GHSV of 80 h^−1^ at a reaction temperature of 220 °C. The same catalyst was subjected to a long-term performance test for 85 h under the conditions of a C_2_H_2_/HCl volume ratio of 1:1.2, a reaction temperature of 220 °C, and a C_2_H_2_ space velocity (GHSV) of 100 h^−1^. The conversion of acetylene (XA) was calculated as follows:

$$X_{A}=\frac{\varepsilon_{A0}-\varepsilon_{A}}{\varepsilon_{A0}}\times 100\%$$

where $\varepsilon_{A0}$ is the volume fraction of acetylene in the raw gas and $\varepsilon_{A}$ is the volume fraction of remaining acetylene in the product gas.

## 3. Characterizations

Scanning electron microscopy (SEM), transmission electron microscopy (TEM), X-ray diffraction (XRD), Raman, X-ray photoelectron spectrometer (XPS), C_2_H_2_ temperature-programmed desorption (C_2_H_2_-TPD), thermogravimetric analysis (TGA) and N_2_ adsorption/desorption experiments were conducted to investigate the physicochemical properties of the as-prepared samples. The details of the characterization methods are described in the Supplementary Information.

## 4. Results and Discussion

**Scheme 1:**
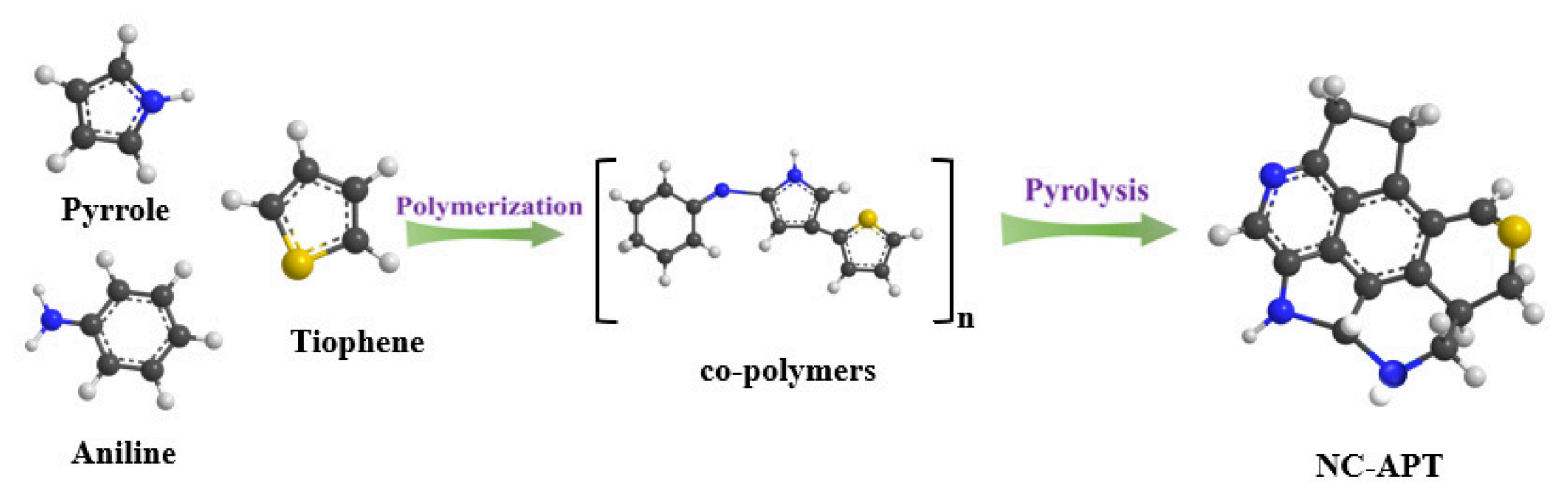
Schematic illustration of the preparation of NC-APT.

As shown in Scheme 1, NC-APT was prepared via the co-polymerization of pyrrole and aniline, mediated by thiophene. This co-polymerization process could introduce more defects compared with the mono-polymerization process due to the structure mismatch between the distinct monomer units. Meanwhile, the presence of the thiophene monomer could provide steric hindrance, disrupting the polymerization regularity. An improvement in catalytic activity is anticipated, benefiting from the defective structure of NC-APT. For comparison, NC-A, NC-P and NC-AP were also prepared via the direct carbonization of PANI, PPY and copolymerized PANI and PPY, respectively (see details in the experimental part).

**Figure 1:**
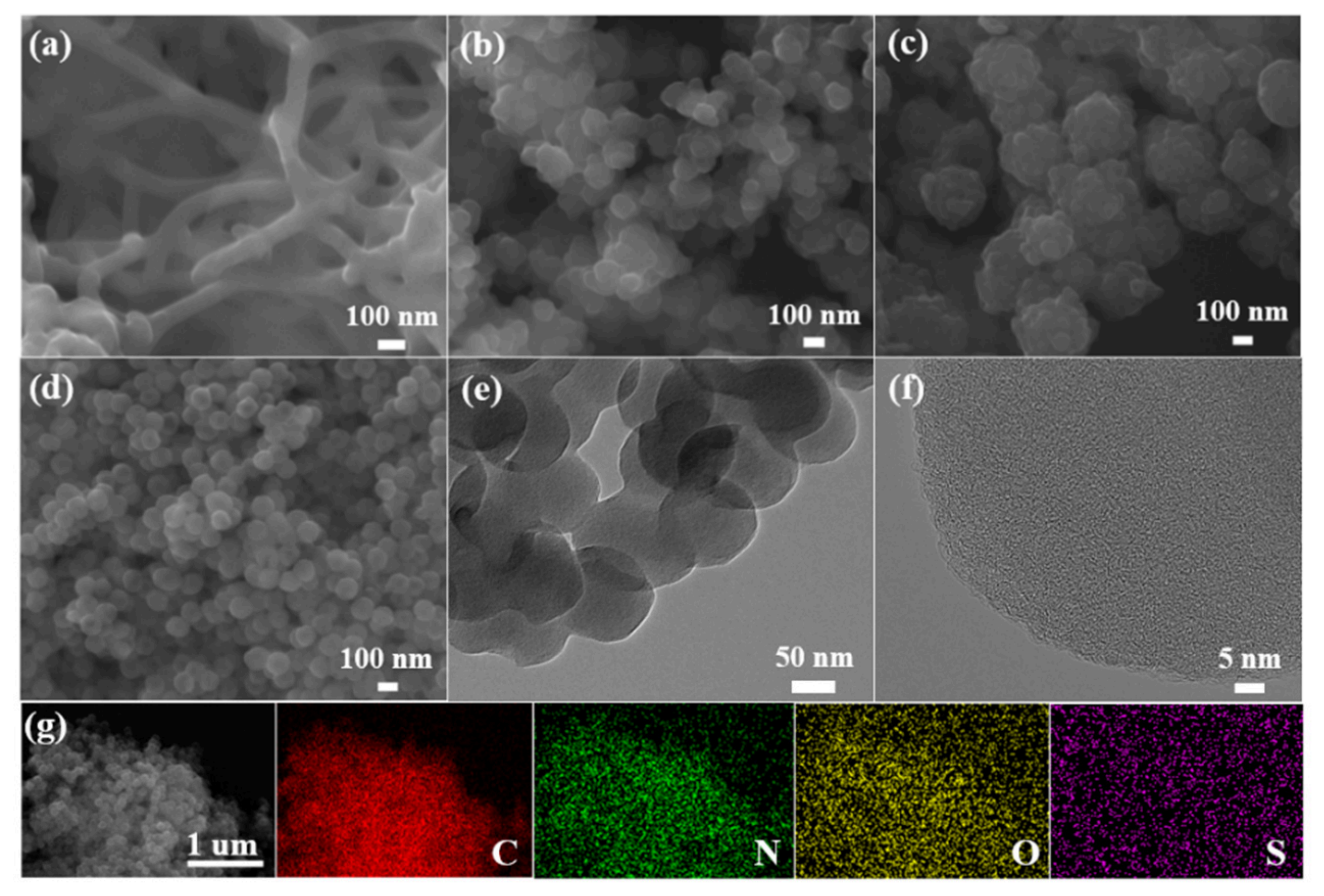
SEM images of (**a**) NC-A, (**b**) NC-P, (**c**) NC-AP, and (**d**) NC-APT, (**e**) TEM and (**f**) HR-TEM images, and (**g**) EDS elemental mapping of NC-APT.

By SEM and TEM characterizations, it was found that aniline and pyrrole form linear and spherical structures, respectively, through self-polymerization (Figure 1a,b). The co-polymerization of aniline and pyrrole yields stable, irregular spherical particles with a rougher surface (Figure 1c). The average particle size of NC-APT is determined to be about 107 nm (Figure 1d), which is much smaller than that of NC-AP. In comparison, the obtained NC-APT demonstrates an inter-connected ball-like structure with a smooth surface. This indicates that the introduction of thiophene could interfere with and faster the co-polymerization process to reduce the polymer size. HR-TEM characterization (Figure 1e,f) confirms the amorphous character of the NC-APT material. The SEM and the corresponding EDS elemental analysis reveal a uniform distribution of C, O, N and S, suggesting successful N-doping and S-incorporation (Figure 1g).

**Figure 2:**
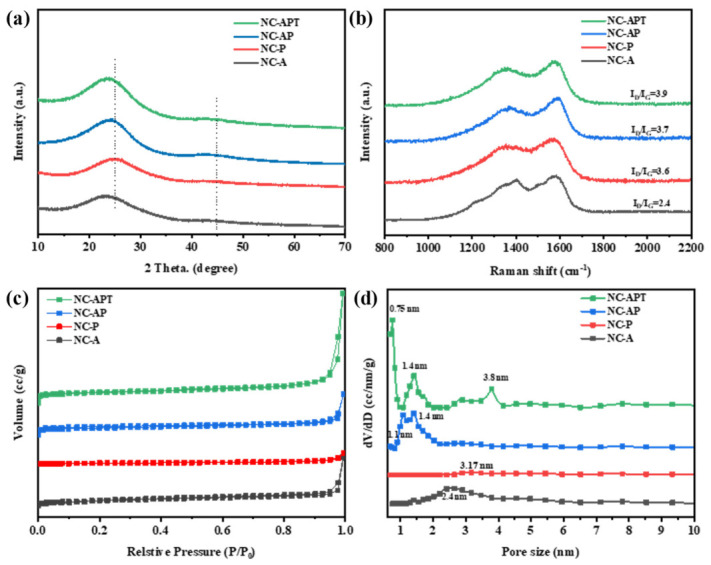
(**a**) XRD patterns, (**b**) Raman spectra, (**c**) N_2_-sorption isotherm curve and (**d**) NL-DFT pore size distributions of NC-A, NC-P, NC-AP, NC-APT.

XRD patterns in Figure 2a show distinct characteristic peaks at 2θ value of 25° and 43°, corresponding to the (002) and (101) planes of the carbon material, respectively. These broad XRD peaks indicate low graphitization, consistent with the HR-TEM results. It is interesting to note the shift in the (002) diffraction peak toward lower angles for NC-APT, which indicates an increase in interlayer distance due to the defect introduction [37]. The Raman spectra display two characteristic peaks at 1350 cm^−1^ and 1580 cm^−1^, representing disordered carbon and graphitized carbon, respectively. A higher ratio of the D band to the G band (I_D_/I_G_) represents a lower graphitization of the carbon material [38]. To process the Raman data, a baseline subtraction was applied, and a Gaussian function was used for the fitting of the D and G peaks, and the information of I_D_/I_G_ was obtained by dividing the area of the D peak by the area of the G peak. As shown in Figure 2b and Table 1, the I_D_/I_G_ values follow NC-APT (3.9) > NC-AP (3.7) > NC-P (3.6) > NC-A (2.4). The error bars of the Raman results are demonstrated in Figure S3, which confirms the results reliability. This sufficiently proves the efficacy of implanting defects via the co-polymerization methodology, which will provide more active sites for reactant activation.

From the N_2_ sorption isotherms in Figure 2c, a slight N_2_ adsorption at a low relative pressure (P/P_0_ < 1) can be seen, indicating the presence of micropores. Meanwhile, a slight hysteresis loop occurs in the middle relative pressure region, suggesting the existence of mesopores [39]. The hierarchical pore structure of the samples is well in accordance with the NL-DFT pore size distribution analysis (Figure 2d). The mean pore size for NC-APT is centered at 0.75 nm and 1.4 nm, while the average pore size of NC-AP is 1.4 nm, and the average pore size of NC-A and NC-P is around 2.4–3 nm. The possible reason for the pore size reduction in NC-APT may be that the release of S compounds during the pyrolysis process facilitates the further creation of micropores. The specific surface area for NC-APT is 375.7 m^2^·g^−1^, obviously higher than those of NC-AP (295.0 m^2^·g^−1^), NC-A (286.4 m^2^·g^−1^) and NC-P (169.0 m^2^·g^−1^) (Table 1). It should be noted that the standard error for the specific surface area test is within 10 m^2^·g^−1^. This result remarkably manifests the highly porous structure of NC-APT, which is pivotal for facilitating active site exposure and promoting mass transfer.

The surface heteroatom-doping information was characterized by XPS. From Table 2 and Figure S1, it can be seen that NC-APT shows the highest N doping amount of 14.4%. However, NC-APT has an ultra-low S doping content of 0.1%. This result suggests that most of the S atoms were released during the calcination process, which may be in the form of SO_x_, as supported by a previous report [40]. This process could help remove O atoms, therefore preventing the formation of NO_x_ and leading to the preservation of N dopant atoms in the framework. Furthermore, the amount of pyrrolic N and pyridinic N, which are named as edge-N, is calculated to be as high as 81% for NC-APT, exceeding that of other samples. This proves both increased active site numbers and enhanced active site exposure for the co-polymerized sample, embodying its potential for the catalytic application as a high-performance metal-free catalyst.

**Figure 3:**
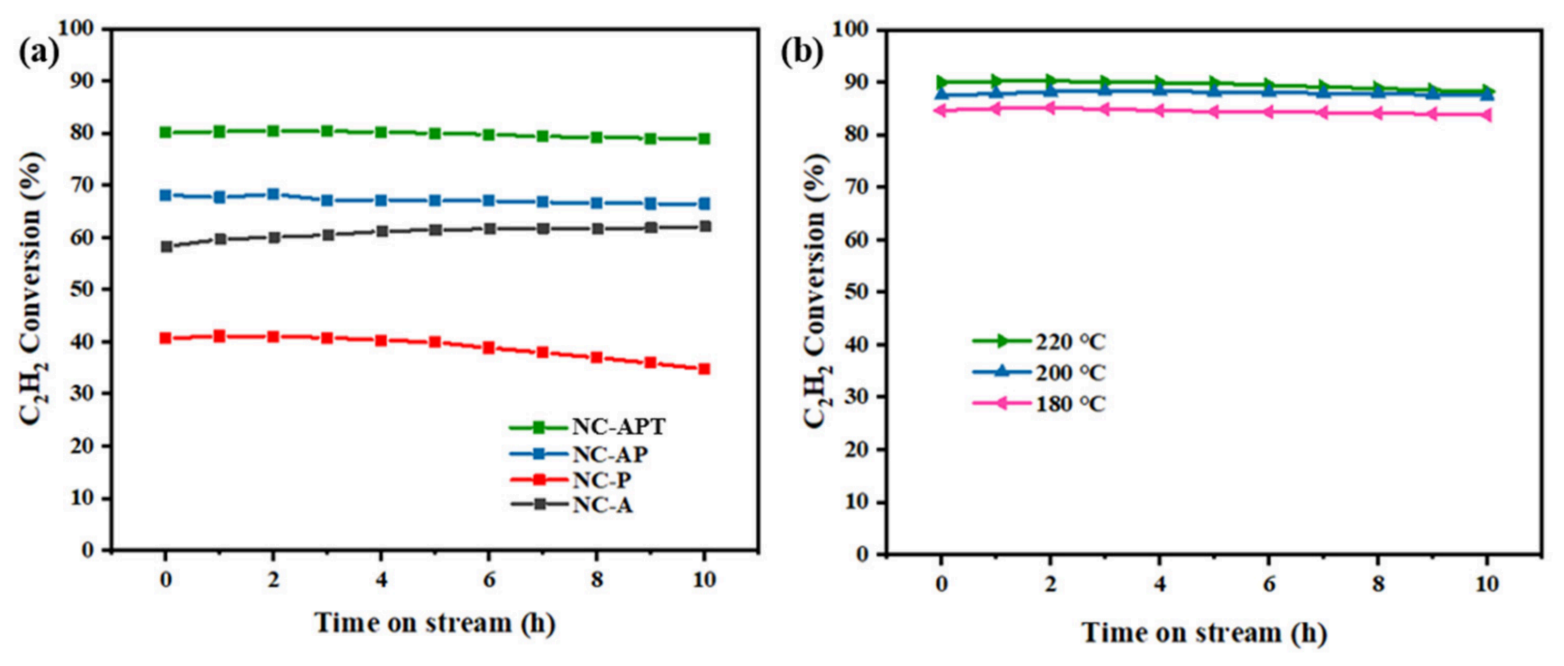
(**a**) Activity evaluation of NC-A, NC-P, NC-AP and NC-APT under reaction conditions of acetylene GHSV = 120 h^−1^, V (C_2_H_2_): V (HCl) = 1:1.2, T = 200 °C, (**b**) acetylene conversion as a function of reaction temperature for NC-APT under acetylene GHSV of 80 h^−1^, V (C_2_H_2_): V (HCl) = 1:1.2.

By evaluating the catalytic performance for acetylene hydrochlorination, it can be seen that the catalytic activity follows NC-APT > NC-AP > NC-A > NC-P (Figure 3a). A high acetylene conversion of 80% was achieved over NC-APT at an acetylene GHSV of 120 h^−1^ and a reaction temperature of 200 °C. Figure S4 demonstrates the error bars for the acetylene conversion of the catalysts, which verifies the reliability of the test results. To further elevate the reactivity, the influence of reaction temperature was optimized at a lower acetylene GHSV of 80 h^−1^. As shown in Figure 3b, the catalytic activity increases with the rise in reaction temperature. NC-APT delivers an acetylene conversion of 92% at 220 °C and the performance is stable over 10 h. We further evaluated the acetylene conversion rate (acetylene conversion per gram of catalyst per hour) and compared it with reported catalysts. As shown in Table S1, NC-APT exhibits a conversion rate of 0.85 mol·g_cat_^−1^·h^−1^, which is one of the most active metal-free catalysts for the acetylene hydrochlorination reaction. The stability of the NC-APT catalyst was evaluated under harsh conditions with an acetylene GHSV of 100 h^−1^ and a reaction temperature of 220 °C. As the results shown in Figure S5, the acetylene conversion decreased by 7% after 85 h of continuous reaction, which may be due to carbon coking on the catalyst surface [41]. As per our previous report, the deactivated metal-free catalyst could be efficiently recovered by high-temperature treatment with ammonia to remove the coked carbon and preserve the doped nitrogen [42]. This adequately proves the excellent metal-free catalytic performance of NC-APT for non-mercury catalysis of the vinyl chloride production.

**Figure 4:**
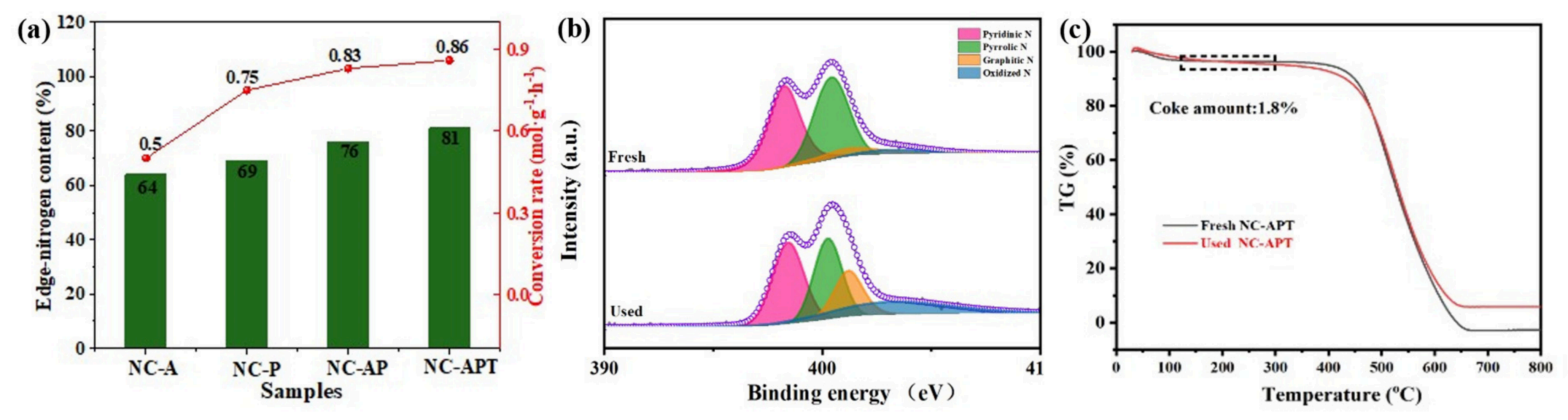
(**a**) The relationship graph between the exposed edge-N content and activity, (**b**) C_2_H_2_-TPD for NC-APT, NC-AP, NC-P, NC-A, (**c**) TG of fresh and used NC-APT (the dashed square in the figure indicates the mass loss corresponding to the coked carbon).

To elucidate the structure–function relationship, we made a co-relation analysis of the acetylene conversion rate with the exposed edge-N content. Specifically, the exposed edge-N content was calculated by multiplying the specific surface area by the catalyst mass (0.8 g) and with the XPS-derived edge-N (the sum of pyrrolic N and pyridinic N) percentage (Figure S2). From Figure 4a it can be seen that the increase in activity is closely corelated with the augmentation of edge-N numbers. For metal-free catalysis in the acetylene hydrochlorination reaction, acetylene activation is commonly considered to be the rate-determining step [41]. The acetylene activation ability was therefore evaluated by acetylene TPD analysis. As the result show in Figure 4b, the acetylene desorption amount follows: NC-APT > NC-AP > NC-A > NC-P. This verifies a higher acetylene adsorption and activation capability for the NC-APT catalyst, which may be due to its enriched edge-N dopants and also the possible contribution of minor S incorporation. Moreover, stability is also a key factor for the industrial process. Carbon coking is deemed to be one of the main reasons causing carbon catalyst deactivation [42]. We therefore performed TG analysis to shed light on the carbon deposition. As shown in Figure 4c and Figure S3, a slight carbon coke amount of about 1.8% is formed on the reacted NC-APT catalyst, which is determined by the mass change within the temperature range of 50 °C to 600 °C. This value is lower than those of NC-AP (3.6%), NC-A (3.8%) and NC-P (4.1%). This result proves the robustness of the catalyst, which shows great promise for practical application.

## 5. Conclusions

In the present work, an effective co-polymerization methodology was developed to produce a highly porous NC catalyst. By leveraging the size mismatch and steric hindrance of the pyrrole, aniline and thiophene monomers, a highly porous structure with enriched edge-N sites was engineered. Excellent catalytic activity was achieved, with an acetylene conversion of 92% at an acetylene GHSV of 80 h^−1^ and a reaction temperature of 220 °C, which is one of the highest-performing metal-free catalysts. This study offers meaningful insights into the pore structure and surface orchestration of the NC catalyst for promoting the replacement of mercury chloride catalysts in the acetylene hydrochlorination reaction.

## Figures and Tables

**Table 1:** Graphitization and pore structure information of the catalysts.

Sample	I_D_/I_G_^a^	Specific Surface Area/m^2^·g^−1^	V_total_^b^/cm^3^·g^−1^
NC-A	2.4	286.4	0.62
NC-P	3.6	169.0	0.14
NC-AP	3.7	295.0	0.52
NC-APT	3.9	375.7	1.44

^a^ The peak intensity ratio of the D band to the G band in Raman spectroscopy. ^b^ The total pore volume calculated at P/P_0_ of 0.99420.

**Table 2:** Surface information of the catalysts.

Sample	Element Composition (%)				N Species Content (%)			
	C	N	O	S	Pyridinic N	Pyrrolic N	Graphitic N	Oxidized N
NC-P	81.7	11.2	7.1	-	38	26	26	10
NC-A	87.5	9.5	2.9	-	42	32	17	8
NC-AP	83.7	11.5	4.9	-	39	37	13	11
NC-APT	82.8	14.4	2.7	0.1	41	40	10	9

## Data Availability

The original contributions presented in this study are included in the article/Supplementary Material. Further inquiries can be directed to the corresponding authors.

## Supplementary Materials

The following supporting information can be downloaded at: https://www.mdpi.com/article/10.3390/nano16090568/s1, Table S1. Catalytic activity comparison with reported catalysts. Figure S1. XPS spectra of NC-APT, NC-AP, NC-P and NC-A. Figure S2. Deconvolution of N1s XPS spectra of NC-APT, NC-AP, NC-P, NC-A. Figure S3. The ID/IG values of NC-APT, NC-AP, NC-P and NC-A with error bars. Figure S4. Acetylene conversion for NC-APT NC-AP, NC-P and NC-A with error bars. The reaction conditions: acetylene GHSV of 100 h^−1^, V (C2H2): V (HCl) = 1:1.2, T = 200 °C. Figure S5. Activity evaluation of NC-APT under reaction conditions of acetylene GHSV = 100 h^−1^, V (C2H2): V (HCl) = 1:1.2 and reaction temperature of 220 °C. Figure S6. TG of (a) NC-AP, (b) NC-P and (c) NC-A. References [22,43,44,45,46,47,48] are cited in the Supplementary File.
