# Supplementary material for: Engineering of Edge-Enriched Nitrogen-Doped Porous Carbon as a High-Performance Metal-Free Catalyst for Acetylene Hydrochlorination
Source: Nanomaterials (Basel). 2026 May 6;16(9):568. doi: 10.3390/nano16090568 (PMC13164920; doi:10.3390/nano16090568)
Supplement: Supplementary file 1 [file nanomaterials-16-00568-s001.zip › nanomaterials-4267080-supplementary.pdf]

Supporting Information

Engineering of Edge-Enriched Nitrogen-Doped Porous Carbon as a High-Performance Metal-Free Catalyst for Acetylene Hydrochlorination

Zhenzhen Zhang [1,†], Dashuai Zhang [2,†], Yalei Hao [1,†], Guangzong Fang [3], Xingyun Li [1,*] and Jian Qi [4,*]

[1] Institute of Materials for Energy and Environment, College of Materials Science and Engineering, Qingdao University, Qingdao 266071, China

[2] Shandong Provincial Key Laboratory of Monocrystalline Silicon Semiconductor Materials and Technology, College of Chemistry and Chemical Engineering, Dezhou University, Dezhou 253023, China

[3] State Key Laboratory of Catalysis, Dalian Institute of Chemical Physics, Chinese Academy of Sciences, Dalian 116023, China

[4] State Key Laboratory of Biopharmaceutical Preparation and Delivery, Institute of Process Engineering, Chinese Academy of Sciences, Beijing 100190, China

* Correspondence: xingyun_2008@sina.cn (X.L.); jqi@ipe.ac.cn (J.Q.)

† These authors contributed equally to this work.

Material characterizations

XRD was performed using Rigaku Ultima IV X (Japan Electron Optics Laboratory Co., Ltd., Tokyo, Japan), with Cu Kα(=0.154178nm) as radiation source. The scanning range was from 10 to 90, and the scan rate was 5° min^{-1}. Field emission scanning electron microscope (FE-SEM) images were examined by a JEOL JSM-7800F (Japan Electron Optics Laboratory Co., Ltd., Tokyo, Japan). Transmission electron microscope (TEM) images were recorded on a JEOL JEM-2100 (Japan Electron Optics Laboratory Co., Ltd., Tokyo, Japan). Low-temperature N_2 adsorption/desorption experiments were performed using Quanta Autosorb iQ3 (Quantachrome Instruments, Florida, USA). The catalyst was pretreated at 250 °C for 10 h under vacuum, followed by N_2 adsorption using liquid nitrogen. The specific surface area was calculated using the BET (Brunauer-Emmett-Teller) method. Pore size distribution was determined using the NL-DFT (Non-Localized Density Functional Theory) method. The micropore volume was calculated using the t-Plot method. The pore volume was obtained from the adsorption amount at P/P_0= 0.9997. Surface elemental analysis and chemical bonding composition of the sample were carried out. using Thermo Fisher ESCALAB 250Xi (Thermo Fisher, Shanghai, China). The X-ray source used was Al Ka (1486.6 eV) with a working voltage of 10 kV and current of 5 mA. C_2H_2-TPD experiments were performed a Chembet Pulsar-type chemisorption analyzer (Quanta chrome, Florida, USA), coupled with a mass spectrometer. The catalyst was first degassed at 80 °C under an Ar atmosphere (20 mL·min^{-1}) for 30 minutes, followed by exposure to a 10% C_2H_2/Ar mixture (20 mL·min^{-1}) at 200 °C for 1 h. The catalyst was subsequently flushed with Ar (20 mL·min^{-1}) for 1 hour to remove physically adsorbed C_2H_2. Then, the temperature was increased at a rate of 10 °C·min^{-1} from 50 °C to 350 °C under Ar atmosphere, and the C_2H_2 signals (m/e= 26) were recorded. Thermogravimetric analysis (TGA) was performed on the samples using a simultaneous thermal analyzer (Model TGA/DSC 2, Mettler-Toledo International Inc., Greifensee, Switzerland) to detect coke deposition on the samples. The experiment was conducted in an air atmosphere with a flow rate of 30 mL·min^{-1}, at a heating rate of 10 °C·min^{-1}, and the temperature was increased from

35 °C to 600 °C.

Table S1. Catalytic activity comparison with reported catalysts.

Sample	[a] C_2H_2 conversion rate ($mol \cdot g_{cat}^{-1} \cdot h^{-1}$)	C_2H_2 Conversion (%)	V_{HCl}: V_{C2H2}	C_2H_2 GHSV (h^{-1})	Reaction temperature (°C)	Ref.
NC-APT	0.85	90.2	1.20	80 h^{-1}	220	This work
5#g-C_3N_4	0.51	76.5	1.15	50 h^{-1}	180	[1]
1#PANI-AC	0.31	72.3	1.17	36 h^{-1}	180	[2]
AC-n-U500	0.66	75.0	1.20	100 h^{-1}	180	[3]
SN-Carbon	0.31	82.4	1.15	50 h^{-1}	180	[4]
C1100	0.61	95.4	1.15	36 h^{-1}	180	[5]
PAN-400	0.24	76.0	1.20	30 h^{-1}	200	[6]
SBNC-800	0.27	99.0	1.20	30 h^{-1}	220	[7]

[a] The catalytic performance was evaluated by TOF, which was calculated using the following equation

$$TOF = n_{C2H2} / (t \times n_{cat})$$

Where n_{cat} is the mass of catalyst, t is the reaction time, and n_{C2H2} is the amount of converted C_2H_2.

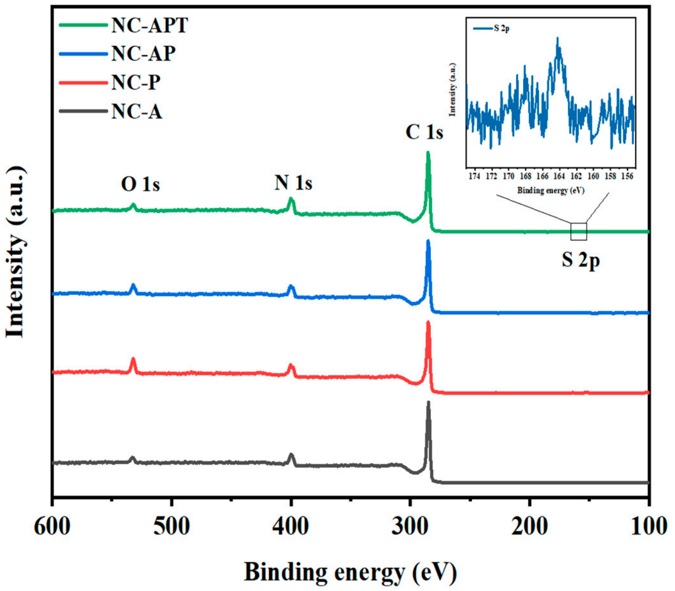

Fig. S1. XPS spectra of NC-APT, NC-AP, NC-P and NC-A.

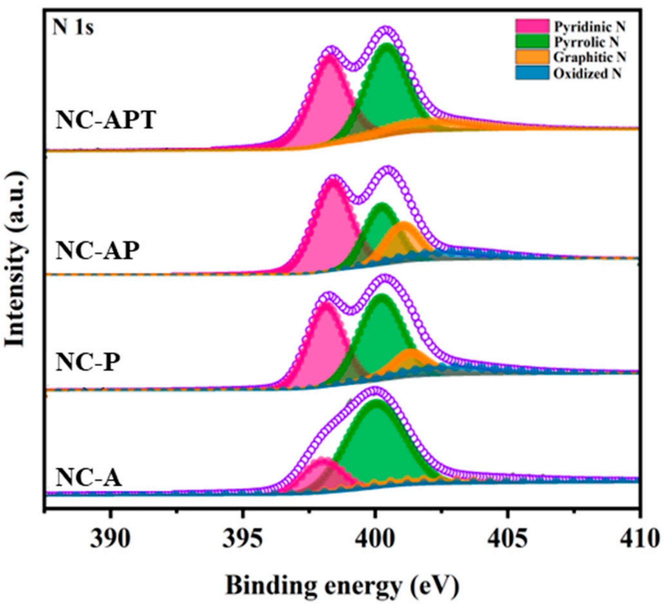

Fig. S2. Deconvolution of N1s XPS spectra of NC-APT, NC-AP, NC-P, NC-A.

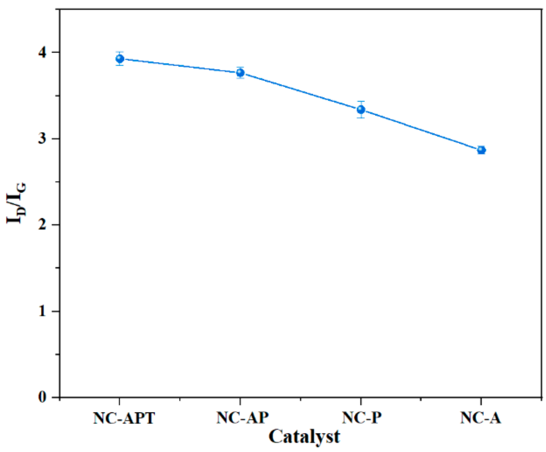

Fig. S3. The I_D/I_G values of NC-APT, NC-AP, NC-P and NC-A (The error line represents the standard error of the average value)

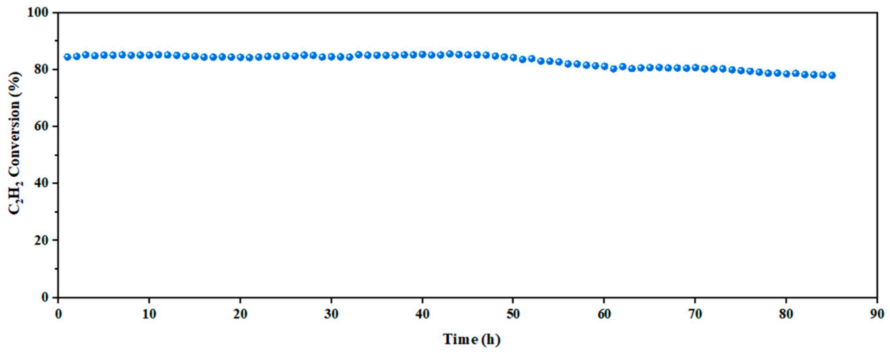

Fig. S4. Activity evaluation of NC-APT under reaction conditions of acetylene GHSV = 100 h^{-1}, V (C_2H_2): V (HCl) = 1:1.2, T=220 °C

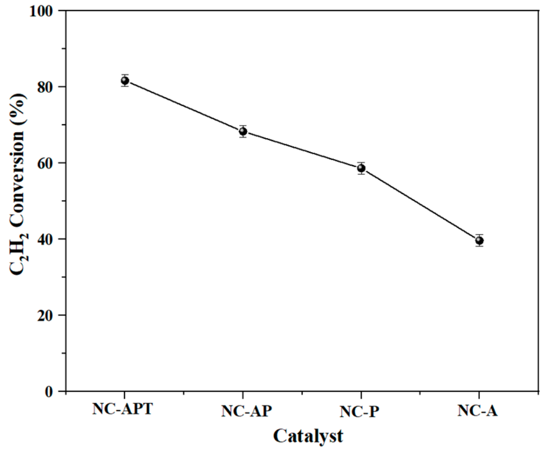

Fig. S5. Initial acetylene conversion as a function of reaction temperature for NC-APT NC-AP, NC-P and NC-A under acetylene GHSV of 100 h^{-1}, V (C_2H_2): V (HCl) = 1:1.2,T=200 °C (The error line represents the standard error of the average value)

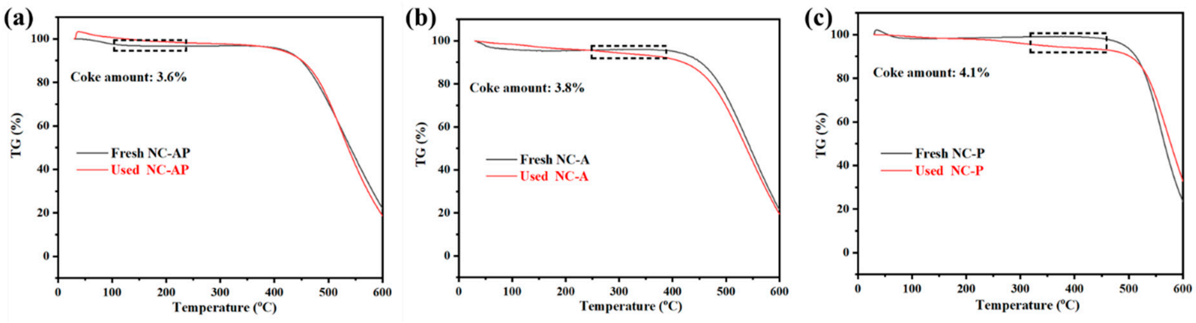

Fig. S6. TG of (a) NC-AP, (b) NC-P and (c) NC-A.

References

[1] Li, X.; Wang, Y.; Kang, L.; Zhu, M.; Dai, B. A novel, non-metallic graphitic carbon nitride catalyst for acetylene hydrochlorination. Journal of Catalysis **2014**, *311*, 288-294.

[2] Zhang, C.; Kang, L.; Zhu, M.; Dai, B. Nitrogen-doped active carbon as a metal-free catalyst for acetylene hydrochlorination. RSC Advances **2015**, *5*, 7461-7468.

[3] Zhang, T.; Zhao, J.; Xu, J.; Xu, J.; Di, X.; Li, X. Oxygen and nitrogen-doped metal-free carbon catalysts for hydrochlorination of acetylene. Chinese Journal of Chemical Engineering **2016**, *24*, 484-490.

[4] Wang, J.; Zhao, F.; Zhang, C.; Kang, L.; Zhu, M. A novel S, N dual doped carbon catalyst for acetylene hydrochlorination. Applied Catalysis A: General **2018**, *549*, 68-75.

[5] Wang, J.; Gong, W.; Zhu, M.; Dai, B. Effect of carbon defects on the nitrogen-doped carbon catalytic performance for acetylene hydrochlorination. Applied Catalysis A: General **2018**, *564*, 72-78.

[6] Qiao, X.; Zhao, C.; Zhou, Z.; Guan, Q.; Li, W. Constructing Pyridinic N-Rich Aromatic Ladder Structure Catalysts from Industrially Available Polyacrylonitrile Resin for Acetylene Hydrochlorination. ACS Sustainable Chemistry & Engineering **2019**, *7*, 17979-17989.

[7] Shen, Z.; Liu, Y.; Han, Y.; Qin, Y.; Li, J.; Xing, P.; Jiang, B. Nitrogen-doped porous carbon from biomass with superior catalytic performance for acetylene hydrochlorination. RSC Advances **2020**, *10*, 14556-14569.
